# One-pot construction of Cu and O co-doped porous g-C_3_N_4_ with enhanced photocatalytic performance towards the degradation of levofloxacin†

**DOI:** 10.1039/c9ra02411e

**Published:** 2019-07-02

**Authors:** Feng Li, Peng Zhu, Songmei Wang, Xiuquan Xu, Zijun Zhou, Chundu Wu

**Affiliations:** Affiliated Hospital of Jiangsu University Zhenjiang 212001 China; School of Pharmacy, Jiangsu University Zhenjiang 212013 China xxq781026@ujs.edu.cn; School of Environment and Safety Engineering, Jiangsu University Zhenjiang 212013 China wucd@ujs.edu.cn

## Abstract

Low visible light response and rapid recombination of photogeneration charge carriers have always been the main factors limiting the advanced application of g-C_3_N_4_ (CN). Element doping has been confirmed to be an efficient method to improve the photocatalytic performance of CN. Here, a series of Cu and O co-doped porous g-C_3_N_4_ (Cu/O-PCN) nanomaterials were successfully fabricated by a facile one-pot thermal polymerization approach for the first time. Compared to pure CN, the resulting Cu/O-PCN exhibited remarkably enhanced visible-light-driven photocatalytic activity towards levofloxacin (LEVO) degradation. The optimized sample of 0.5% Cu doped (Cu/O-PCN-3) presented the highest degradation rate constant of 0.0676 min^−1^, which was about 6.2 times higher than that of CN. Furthermore, a substantial decrease in the residual toxicity against *E. coli* was observed after photocatalytic degradation treatment. The superior photocatalytic performance of Cu/O-PCN was mainly attributed to the synergistic advantages of stronger visible light response, larger specific surface area, and the more effective separation and transfer of photogenerated charge carriers. Moreover, according to the trapping experiments, ·O_2_^−^ and h^+^ were the major oxygen active species in the photocatalytic degradation process. Finally, the possible enhanced photocatalytic mechanism over Cu/O-PCN was proposed.

## Abstract 2

Cu and O co-doped porous g-C_3_N_4_ with outstanding photocatalytic degradation of levofloxacin performance was synthesized by a one-pot thermal polymerization approach.

## Graphical abstract

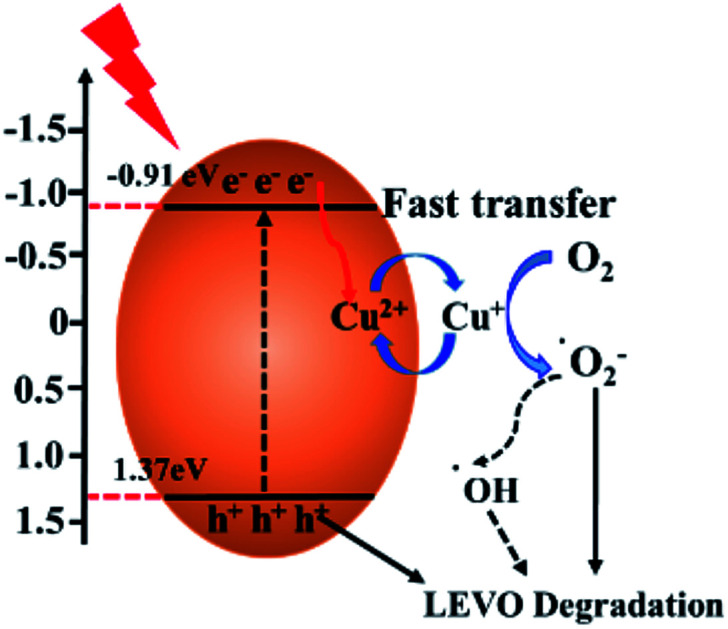

## 1. Introduction

Levofloxacin (LEVO), a typical fluoroquinolones antibiotic, has been widely used in the treatment of various bacterial infectious diseases, benefitting from its broad activity spectrum and good bioavailability.^1^ Thus, frequent occurrence of LEVO has been detected in wastewater due to its overuse and inherent resistance to biodegradability characteristics.^2,3^ However, the increasing emergence of resistant bacteria caused by LEVO will pose a serious threat to the ecosystems, especially to human health. Therefore, it is necessary to seek an effective and environmentally friendly way to remove LEVO from wastewater. In recent years, some conventional advanced oxidation techniques including electron-Fenton, H_2_O_2_/UV, ultrasound (US)/H_2_O_2_ have been developed to deal with LEVO residues in wastewater.^4–6^ Compared to these techniques, photocatalytic degradation based on a visible-light-driven semiconductor has been generally accepted to be the most promising and green technology to completely remove these antibiotic residues because of its stable chemical and physical properties, high photocatalytic efficiency, economic and ecofriendly characteristics,^7,8^ and several of efficient photocatalysts, such as Ag/AgBr/BiOBr, Bi_2_WO_6_, Nb_2_O_5_/g-C_3_N_4_ have been employed to dispose LEVO residues.^9–11^ In particular, g-C_3_N_4_ received considerable attention owing to its visible light response capability, good photochemical stability, facile preparation and environmental friendliness.^12,13^ Unfortunately, the practical application of pristine g-C_3_N_4_ was still suffered from its intrinsic drawbacks including poor surface area, weak visible light response as well as the rapid recombination rate of charge carries.^14,15^ To date, numerous effective strategies have been devoted to improve the photocatalytic performance of g-C_3_N_4_ including porous-structuring, element doping, dye sensitizing and heterojunction constructing.^16–19^ Among these strategies, element doping is proved to be a simple and effective approach to enhance g-C_3_N_4_ photocatalytic activity by modifying its band structure, enhancing the visible light absorption, promoting the electron–hole pairs separation, and prolonging the lifetime of charge carriers, which are all necessary in photocatalytic process.^17,20,21^ Recently, it was more exciting that dual doping of g-C_3_N_4_ by co-doping nonmetal or metal ions could combine the advantages of these single dopants, leading to enhanced photocatalytic activity,^22^ and some co-doped g-C_3_N_4_ based nanomaterials, including I and K, O and Na, P and Mo, Fe and P have been demonstrated to exhibit higher photocatalytic activity than that of single element doping.^23–26^

Herein, we constructed Cu and O co-doped porous g-C_3_N_4_ with superior photocatalytic performance *via* a facile one-pot thermal polymerization method. The photocatalytic degradation of LEVO was carried out to evaluate their photocatalytic activities under visible light irradiation. On the basis of the experimental results, the possible enhanced photocatalytic mechanisms were discussed in detail.

## 2. Experiments

### 2.1. Chemicals

All chemical reagents with analytical grade were purchased from Sinopharm Chemical Factory of China and used without further purification. The deionized water with a resistivity ≥ 18.2 MΩ cm^−1^ was used throughout the experiments.

### 2.2. Synthesis of Cu and O co-doped porous g-C_3_N_4_ nanomaterials

The series of Cu and O co-doped porous g-C_3_N_4_ nanomaterials were prepared simply *via* a one-pot thermal polymerization method using urea as precursor while formic acid and copper nitrate as dopants. In a typical synthesis, 20 g urea, 0.5 mL formic acid and a certain amount of Cu(NO_3_)_2_·3H_2_O were dissolved into 10 mL deionized water for 30 min under ultrasonic treatment, where the amount of Cu(NO_3_)_2_·3H_2_O was 25, 50, and 100 mg, respectively. After that, the mixture was heated at 60 °C under vacuum until water was completely evaporated. Whereafter, the product was placed into a ceramic crucible with a cover and calcined at 550 °C for 2 h at the rate of 5 °C min^−1^ under air atmosphere in a muffle furnace. With cooling to room temperature, the Cu and O co-doped porous g-C_3_N_4_ photocatalysts with different mass ratio of Cu(NO_3_)_2_·3H_2_O to urea (0.125%, 0.25%, 0.5%) were obtained and denoted as Cu/O-PCN-1, Cu/O-PCN-2 and Cu/O-PCN-3, respectively. For comparison, g-C_3_N_4_ was prepared under the same conditions *via* direct calcination of urea and named as CN.

### 2.3. Characterizations

The crystalline phases of as-prepared nanomaterials were recorded on a Bruker D8 Advance X-ray diffractometer equipped with a Cu Kα radiation ranging from 10° to 80° at a scanning rate of 5° min^−1^. The X-ray photoelectron spectrum (XPS) was carried out on an ESCALAB 250 Xi spectrometer using Al Kα X-ray source. The apparent and internal morphologies were presented using a S4800 field emission scanning electron microscope (SEM) and a JEM2100F high-resolution transmission electron microscope (TEM). The elemental mapping was detected by an energy-dispersive X-ray (EDX) spectrometer attached to the TEM. Nitrogen adsorption–desorption isotherms and pore size distributions of samples were measured on a Micromeritics Tristar 3000 analyzer. The UV-vis diffuse reflectance spectroscopy (DRS) was measured on an UV-2600 UV-vis spectrophotometer from 200 nm to 800 nm with BaSO_4_ as a reflectance standard. Photoluminescence and time-resolved fluorescence spectroscopy was performed using a FLS980 steady-state and time-resolved fluorescence spectrometer.

The transient photocurrent and electrochemical impedance spectroscopy (EIS) measurements were performed on an electrochemical workstation (CHI 660C, China) equipped with a 300 W Xe lamp as the visible light source. In the standard three-electrode system, Pt wire, saturated Ag/AgCl and FTO deposited with nanomaterials were served as the counter, reference, and working electrodes, respectively. The working electrodes were assembled by electrophoretic deposition according to the previous study.^25^

### 2.4. Photocatalytic activity evaluation

The photocatalytic activity of as-prepared nanomaterials was evaluated in terms of the degradation of LEVO in aqueous solution. A 300 W xenon lamp equipped with an UV cut-off filter (*λ* > 420 nm) was employed as the visible light source. In a typical process, 50 mg photocatalyst was dispersed into 50 mL of 15 mg L^−1^ LEVO solution. The mixture was kept stirring in dark for 30 min to achieve adsorption–desorption equilibrium. At given time intervals of irradiation, 3 mL of suspension was removed and filtered. The concentration of LEVO was determined by high performance liquid chromatography (HPLC, Agilent 1260) equipped with a UV-vis detector (Agilent G1314F).

### 2.5. Residual toxicity test

The residual toxicity was evaluated the antibiotic activity of reaction solution towards *E. coli* by disc diffusion method.^27^ Detailedly, A 100 μL *E. coli* (DH5α) suspension of 1.0 × 10^7^ CFU mL^−1^ was evenly spread over the surface of the Luria–Bertani solid agar plates, followed by placing sterilized filter papers (6 mm diameter) onto them. And then, 20 μL of initial and treated solutions after 20, 45 and 60 min were poured onto filter papers, respectively. Following of incubation at 37 °C overnight, the inhibition zone diameters around the filter papers were measured.

## 3. Results and discussion

### 3.1. Characterization of as-prepared nanomaterials

**Fig. 1:**
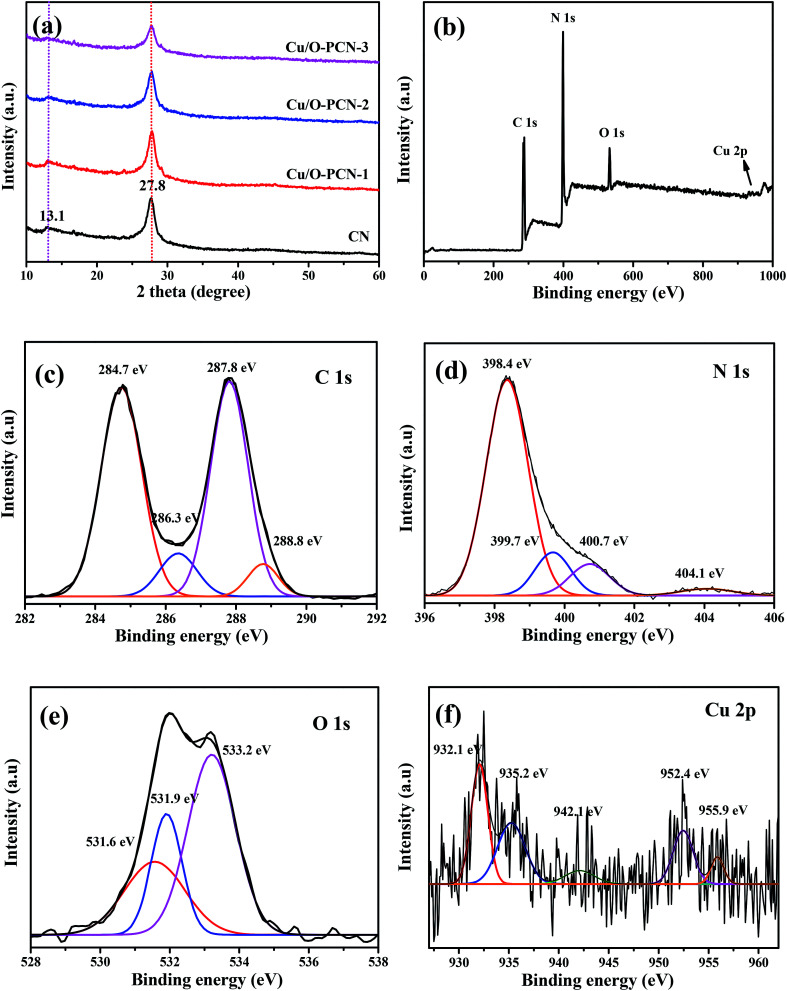
(a) XRD patterns of as-prepared nanomaterials. XPS spectra of Cu/O-PCN-3: (b) survey spectrum and the corresponding high-resolution of (c) C 1s, (d) N 1s, (e) O 1s, and (f) Cu 2p.

The crystal structure of obtained nanomaterials were carefully identified by XRD. Fig. 1a displayed that two distinct peaks located at 2*θ* of about 13.1° and 27.8° could be clearly observed in all samples, which were well indexed to the (100) in-plane structural packing motifs of *s*-triazine ring units and the (002) interlayer stacking of the conjugated aromatic systems of CN (JCPDS no. 87-1526) respectively,^23,28^ indicating that the dopant elements of Cu and O did not change the lattice structure of CN. In addition, these were no peaks of Cu species, such as CuO, Cu_2_O, could be observed in the XRD patterns of Cu/O-PCN samples, signifying the distribution of Cu in the form of single ion. Obviously, compared with CN, the intensity of these two peaks were all gradually decreased and broadened with the increasing Cu dopant, which indicated that the crystallinity of Cu/O-PCN samples are lower than that of CN.

The surface compositions and bonding structure of Cu/O-PCN-3 was investigated by XPS. As shown in Fig. 1b of the survey spectrum, the elements of C, N, and O could be seen clearly with sharp peaks, whereas, a very weak peak attributed to Cu could be observed barely owing to its low doping content. Moreover, the high-resolution spectra of C 1s could be divided into four peaks (Fig. 1c). The peak center at 284.7 eV originates from graphitic carbon (sp^2^ C–C bonds), while the peaks at 287.8 eV corresponds with the sp^2^-hybridized carbon in *s*-triazine ring (N–C=N or C=N).^29^ Two peaks at 286.3 eV and 288.8 eV are derived from the C–O–C and O–C=O groups,^30,31^ which indicates that O atoms are doped into the framework of CN heterocycles by replacing N atoms to form O–C bonds. With regard to the N 1s spectra (Fig. 1d), three N peaks are mainly exhibited, including sp^2^-hybridized N atoms (C–N=C, 398.4 eV), sp^3^ bonded N atoms (N–(C)_3_, 399.7 eV) and the terminal NH_2_ groups (400.7 eV).^32^ The weak peak at 404.1 eV might be ascribed to the feature of π-excitations.^33^ As shown Fig. 1e of O 1s spectrum, the two peaks located at 531.6 and 533.2 eV are corresponded to the adsorbed water and C=O from CO_2_, respectively.^34^ Notably, an obvious peak appeared at 531.9 eV can be ascribed to the C–O–C and N–C–O bonds in the *s*-triazine units by the partial substitution of O with N position.^31,35^ In addition, for the Cu 2p spectra as shown in Fig. 1f, the peaks located at 932.1 and 934.4 eV are corresponded to Cu^+^and Cu^2+^ of Cu 2p_3/2_, whereas at 952.4 and 955.9 eV to Cu^+^and Cu^2+^ of Cu 2p_1/2_, respectively.^36,37^ And the broad weak peak center at 942.1 eV can be ascribed to Cu^2+^ satellite. This result confirms that the Cu exists in Cu/O-PCN nanomaterials with dual oxidation states of Cu^+^ and Cu^2+^.

To further reveal the species of Cu in Cu/O-PCN-3 samples, the FT-IR spectra were carried out. As shown in Fig. S1 (ESI†), the peaks appears at about 576 and 630 cm^−1^ corresponds to the stretching vibration of Cu(ii)–O and Cu(i)–O bonds are not detected in Cu/O-PCN-3,^38,39^ which further confirming that Cu was ionically doped into the porous g-C_3_N_4_ framework.

**Fig. 2:**
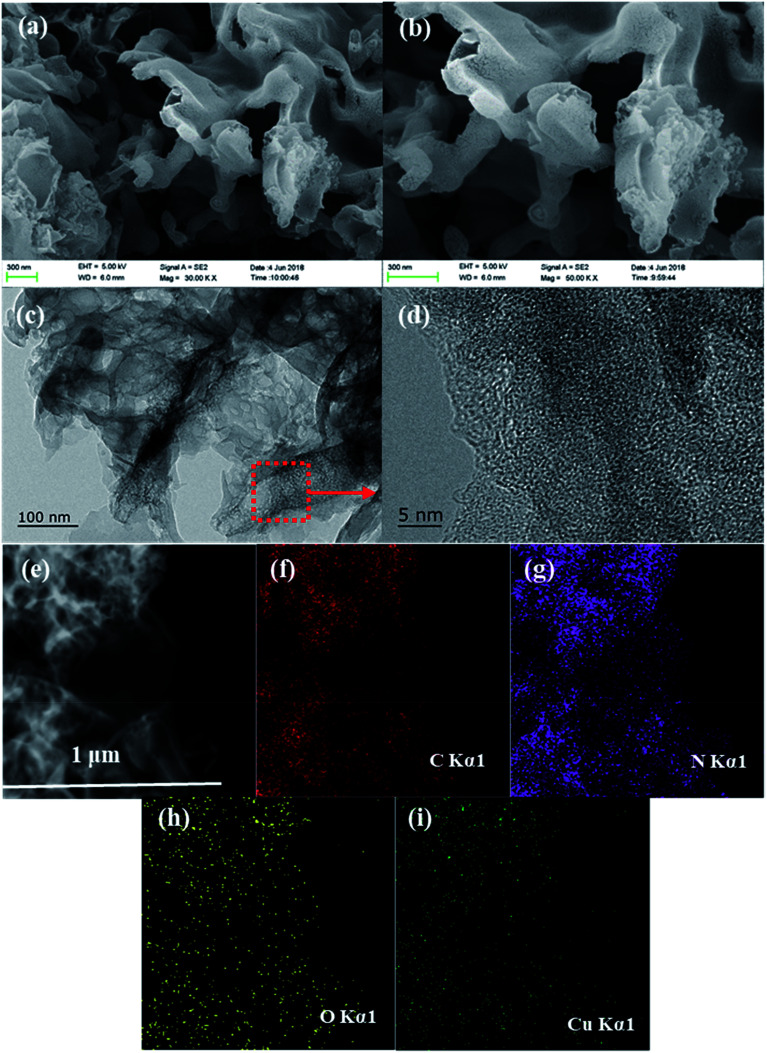
(a and b) SEM and (c and d) TEM images of Cu/O-PCN-3, (e) STEM image of Cu/O-PCN-3 and its corresponding EDS mappings for (f) C, (g) N, (h) O, and (i) Cu.

The surface morphologies of the Cu/O-PCN-3 was observed by SEM and TEM. As shown in Fig. 2a and b, after doping of Cu and O atoms, Cu/O-PCN-3 displayed a noticeable hierarchical nanosheet structure with abundant of irregular in-plane pores distributed on its surface. In addition, from the higher magnification TEM images (Fig. 2c and d), a large amount of holes with the size ranging from several to tens of nanometers were apparently observed on the surface of Cu/O-PCN-3. These porous structure of Cu/O-PCN-3 is probably due to the release of O_2_, CO_2_, NO_2_ and NH_3_ during thermal polymerization the mixtures of urea, formic acid and copper nitrate. It is believed that such structures will provide a large number of active sites, which is very favorable for photocatalytic degradation. Additionally, the EDS elemental mapping images in Fig. 2e–i clearly confirmed that C, N, O and Cu atoms were co-existed and uniformly distributed throughout the surface of Cu/O-PCN-3. These results provide further strong direct evidences of the successful doping of Cu and O elements into the framework of CN.

**Fig. 3:**
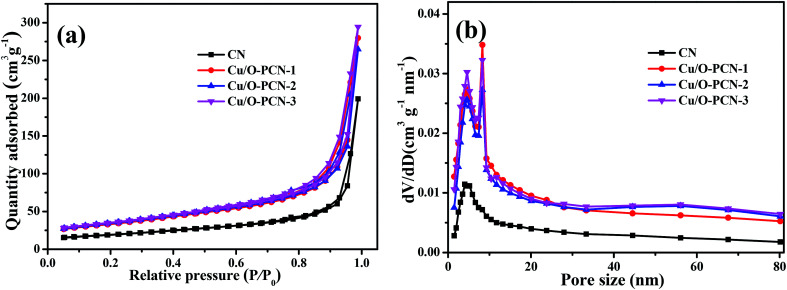
(a) Nitrogen adsorption–desorption isotherms and (b) BJH pore size distribution of as-prepared nanomaterials.

To obtain more about porous properties and specific surface area of the as-prepared nanomaterials, the N_2_ adsorption–desorption isotherms and BJH pore-size distribution curves were measured. As shown in Fig. 3a, all samples exhibited classical type IV adsorption–desorption isotherms with the H_3_ hysteresis loops, suggesting their mesoporous characteristics.^40^ The BET specific surface area (*S*_BET_) of CN was calculated to be 33.68 m^2^ g^−1^. By contrast, these Cu/O-PCN nanomaterials, caused a great enhancement of the *S*_BET_ up to 123.22, 126.56, 135.82 m^2^ g^−1^ with the enhancement of Cu doping. The largest pore volume of Cu/O-PCN-3 was measured as 0.165 cm^3^ g^−1^, which was approximately 3.9 times larger than 0.045 cm^3^ g^−1^ of CN. From the BJH pore size distribution curves (Fig. 3b), compared with CN, Cu/O-PCN revealed relatively broader distribution region with the more prominent pore size diameter centering at about 4.8 and 8.1 nm respectively, which indicated the more mesopores distributing in the basal plane of Cu/O-PCN than that of macropores.^16^ The increased surface area and pore volume of Cu/O-PCN could provide more active sites for both light adsorption and photocatalytic degradation reaction.

**Fig. 4:**
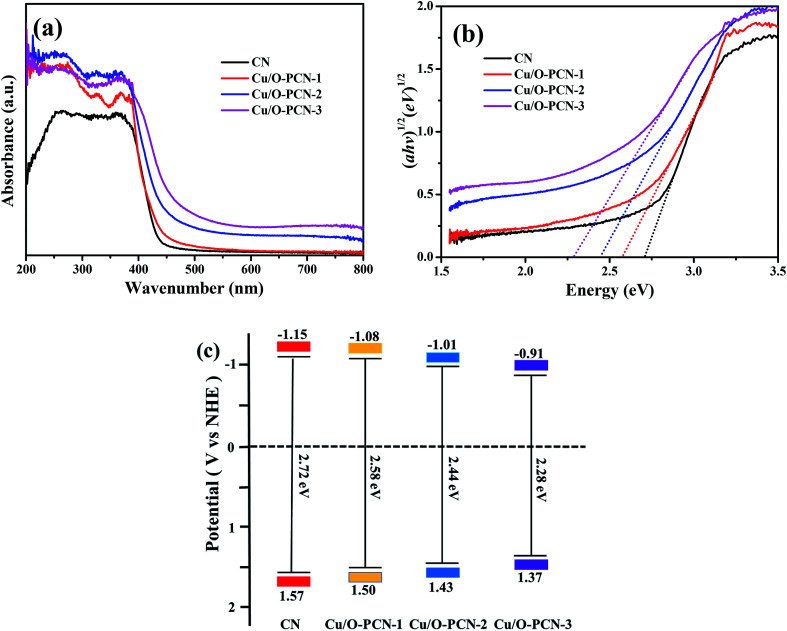
(a) UV-vis diffuse reflectance spectra, (b) Kubelka–Munk spectra and (c) the estimated band positions of as-prepared nanomaterials.

The optical absorption properties of as-prepared nanomaterials were determined by UV-vis DRS. As depicted in Fig. 4a, CN demonstrates an obvious absorption in visible light region at a characteristic absorption edge of around 456 nm. Compared to CN, Cu/O-PCN samples exhibited a gradual red shift as well as a distinct increased absorption over the specified wavelength range with increasing of Cu dopant. According to the Kubelka–Munk method,^41^ these Cu/O-PCN samples also presented gradually narrow band gap located at 2.58, 2.44 and 2.28 eV compared to the 2.72 eV of CN (Fig. 4b). Additionally, the valence band (*E*_VB_) and conduction band (*E*_CB_) edge position of the as-prepared nanomaterials were also calculated according to Mulliken equations as follows,^42^ and summarized in Fig. 4c.*E*_VB_ = *X* − *E*_e_ + 0.5*E*_g_*E*_CB_ = *E*_VB_ − *E*_g_where *X* is the electronegativity of the semiconductor, the *X* value for CN is 4.73 eV.^43^*E*_e_ is the energy of free electron on the hydrogen scale (about 4.5 eV *vs.* NHE).

These results confirm that the Cu and O co-doping can enhance the absorption of visible light, decrease the band gap, thus will harvest more visible light and exhibit higher photocatalytic efficiency.

### 3.2. Photocatalytic activity and mechanism

**Fig. 5:**
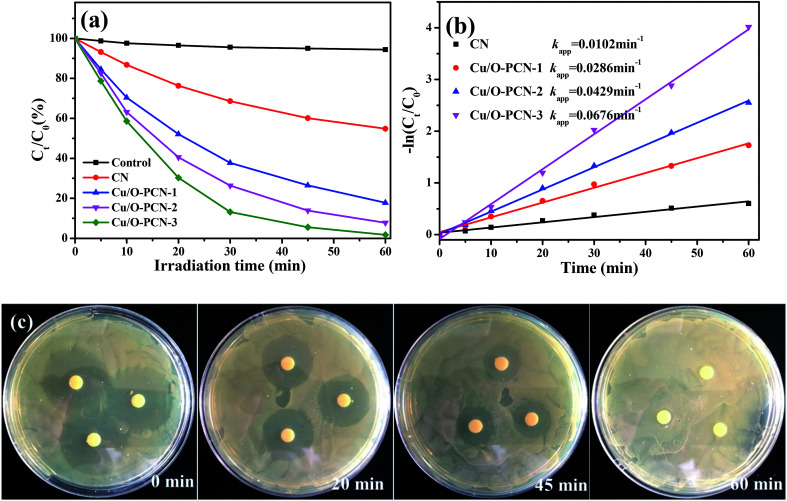
(a) Photocatalytic degradation of LEVO, (b) pseudo first-order kinetic plots of −ln(*C*_*t*_/*C*_0_) for LEVO as a function of visible light irradiation time with as-prepared nanomaterials and (c) the change in the antibacterial activity of LEVO solution during the photocatalytic process by Cu/O-PCN-3.

The photocatalytic performance of as-prepared nanomaterials was investigated by the degradation of LEVO under visible light irradiation. As shown in Fig. 5a, it was obvious that all the Cu/O-PCN samples clearly showed higher photocatalytic degradation activity than pure CN. With increasing of Cu contents, the photocatalytic activity was improved and the Cu/O-PCN-3 nanomaterials displayed the highest degradation rate with almost complete degradation of LEVO in 60 min under visible light irradiation. The plots of −ln(*C*_*t*_/*C*_0_) *vs.* irradiation time are linear (Fig. 5b), suggesting that the photocatalytic degradation of LEVO is well agree with pseudo-first-order model.^44^ And the calculated apparent rate constants (*k* values) were 0.0102, 0.0286, 0.0429, 0.0676 min^−1^ for CN, Cu/O-PCN-1, Cu/O-PCN-2, Cu/O-PCN-3, respectively. In particular, the Cu/O-PCN-3 presented the highest *k* value, which was about 6.6 times higher than that of CN.

The antibacterial activity was measured to evaluate the residual toxicity changes of LEVO solution during photocatalytic treatment. As shown in Fig. 5c, with the progress of photocatalytic degradation, the diameter of inhibition zones of the treated solutions were gradually decreased and finally disappeared after 60 min treatment. These results indicated that the antibacterial functional groups of LEVO were completely destroyed during the Cu/O-PCN-3 photocatalytic degradation process.

**Fig. 6:**
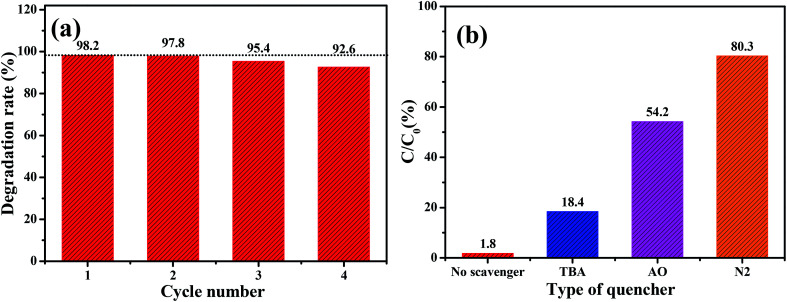
(a) Recycling stability experiments for Cu/O-PCN-3 and (b) active species trapping experiments of Cu/O-PCN-3 during degradation of LEVO.

The stability of Cu/O-PCN-3 was evaluated by recycling the photocatalytic degradation experiments. As shown in Fig. 6a, it can be observed that there is no obvious loss of LEVO degradation after four consecutive experimental runs, suggesting that this sample possesses excellent stability.

To explore the photocatalytic degradation mechanism of Cu/O-PCN-3, the trapping experiments were implemented to determine the main active species in the photocatalytic process. *t*-Butyl alcohol (TBA), ammonium oxalate (AO), and N_2_ were selected as the scavengers for hydroxyl radical (·OH), hole (h^+^) and superoxide radical (·O_2_^−^) respectively.^45–47^ From Fig. 6b, the photocatalytic degradation of LEVO was most obviously inhibited accompanied by the injection with pure N_2_. It indicates that the dissolved O_2_ is the most critical factor for LEVO degradation in the case of Cu/O-PCN-3 which affects the formations of ·O_2_^−^ by reacting with O_2_ directly.^47^ When the addition of AO, the degradation efficiency of LEVO obviously decreased, implying that the h^+^ was also the main active species for LEVO degradation. Instead, when the addition of TBA, the degradation rate of LEVO just slightly decreased, revealing that there were a little ·OH radicals in the photocatalytic system.

**Fig. 7:**
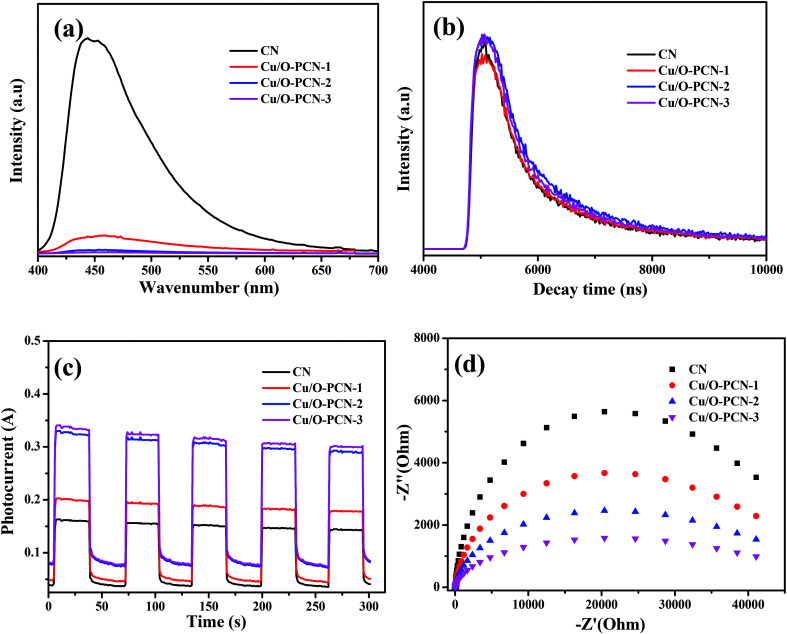
(a) Photoluminescence spectra, (b) time-resolved fluorescence spectra, (c) photo-current response and (d) EIS Nyquist plots for as-prepared nanomaterials.

It is widely accepted that the enhancement of photocatalytic performance of photocatalyst is attributed to the efficient separation and transfer of charge carriers.^48,49^ To achieve the charge carrier separation and transfer characteristics of as-prepared nanomaterials, photoluminescence (PL) spectra and time-resolved fluorescence spectra measurements were carried out. In Fig. 7a, the pure CN presented a strong fluorescence center at around 440 nm, which indicated a high recombination rate of charge carriers.^50^ However, when a certain amount of Cu and O are co-doped, the PL intensity of Cu/O-PCN samples decreased obviously, and the Cu/O-PCN-3 exhibited the lowest intensity of fluorescence, suggesting the lowest recombination rate of charge carriers among the studied nanomaterials. The time-resolved fluorescence spectra of the as-prepared nanomaterials were depicted in Fig. 7b. According to the fitting calculation of the decay spectrum,^51^ the average lifetime markedly increased from 451.82 ns for CN to 517.86, 593.99, 670.36 ns for Cu/O-PCN-1, Cu/O-PCN-2, and Cu/O-PCN-3, respectively. Cu/O-PCN-3 also exhibited the longest lifetimes in all tested samples, demonstrating the highest separation and transfer efficiency of charge carriers, which is favorable for improving photocatalytic activity.

The significantly enhanced charge carrier migration properties of Cu/O-PCN nanomaterials were also evaluated by transient photocurrent and electrochemical impedance spectroscopy (EIS). As shown in Fig. 7c and d, Cu/O-PCN nanomaterials presented much higher photocurrent intensity and smaller arc radius than that of CN, which indicated these Cu/O-PCN nanomaterials had much lower electron-transfer resistance and higher charge transfer efficiency.^52^ Distinctly, the Cu/O-PCN-3 showed the highest photocurrent intensity and smallest arc radius among all the samples, certifying that the suitable Cu and O co-doping can obviously promote the separation and transfer of interfacial charge. The results of photocurrent and EIS analysis are in line with the results of photocatalytic degradation, PL and time-resolved fluorescence analysis, father confirming the advantage of the strategy of no-metal/metal co-doping for improving the photocatalytic performance of CN.

**Fig. 8:**
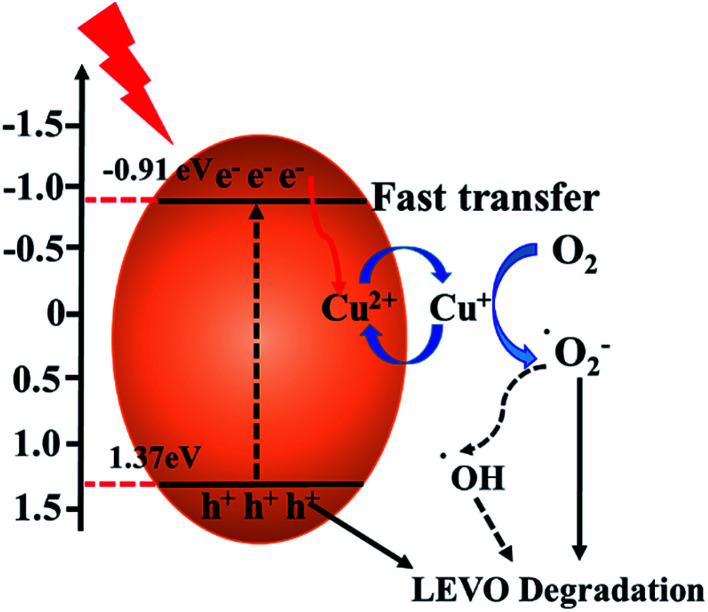
Illustration of the possible charge transfer and separation processes of Cu/O-PCN-3 under visible light irradiation.

Based on the above experimental results, the possible process of photogeneration carrier's transfer and the corresponding enhanced photocatalytic mechanism of Cu/O-PCN-3 for degradation of LEVO was proposed and shown in Fig. 8. The doped O can improve the absorption of Cu/O-PCN-3 in the visible light region by modifying the electronic and microscopic structure through the partial substitution of the N atoms.^53,54^ The doped Cu^2+^/Cu^+^ can serve as a temporary efficient electron capturing centers because of its reduction potential (+0.17 eV/*vs.* NHE) existing between the *E*_CB_ and *E*_VB_ of Cu/O-PCN-3.^42,55^ Under visible light irradiation, the electrons can be excited into the CB of Cu/O-PCN-3, while the holes remain in its VB. The Cu^2+^ species located on the surface of Cu/O-PCN-3 can easily trap the photo-excited electrons and to be reduced to Cu^+^. Owing to its reduction potential more positive than that of O_2_/·O_2_^−^ (−0.33 eV/*vs.* NHE), these freshly formed Cu^+^ will reduce the surficial O_2_ to form ·O_2_^−^, which maybe further react with water to generate ·OH radicals. Simultaneously, Cu^2+^ can be regenerated in this process of cyclic reaction. Thus, this type of self-redox cycle of Cu^2+^/Cu^+^ can significantly promote separation of charge carriers and accelerate the process of interfacial electron transfer. Meanwhile, because of the efficient consumption of electrons, more accumulating holes on the surface of Cu/O-PCN-3 will directly participate in the photocatalytic degradation of LEVO. Unfortunately, this holes cannot oxidize water and OH^−^ to produce ·OH radicals because of its lower oxidation potential of 1.37 eV against H_2_O/·OH (2.27 eV *vs.* NHE) or OH^−^/·OH (1.99 eV *vs.* NHE), respectively. As the result, ·O_2_^−^ and h^+^ were main reactive species for the degradation of LEVO, which was also confirmed by trapping experiments.

## 4. Conclusion

In summary, for the first time, the Cu and O co-doped porous g-C_3_N_4_ (Cu/O-PCN) with excellent photocatalytic property was facilely prepared and applied to degradation of antibiotic pollutants. The XRD patterns, SEM and TEM images along with the adsorption–desorption isotherms confirmed the porous structure of O–Cu-PCN samples, and the XPS analyses verified the doping with Cu at the states of Cu^2+^/Cu^+^. Under visible light irradiation, the Cu/O-PCN samples exhibited enhanced photocatalytic activity for the degradation of levofloxacin than pure CN. Observably, Cu/O-PCN-3 displayed the highest photocatalytic activity: the rate constant reached 0.0676 min^−1^, which was about 6.2 times higher than that of pure CN. The enhanced photocatalytic activity was mainly attributed to the changed intrinsic electronic and band structure of CN by Cu and O co-doping, which results in the efficient visible light response, the high separation and transfer performance of photogenerated charge carriers. Finally, the trapping experiments confirmed that ·O_2_^−^ followed by h^+^ were responsible for the photocatalytic degradation levofloxacin process. This present work demonstrates a simple method for integrating the doping of different elements to achieve excellent visible light driven photocatalytic performance of CN based materials, which will simultaneously enhance the visible light response and charge carrier transfer capacity, thus exhibits great potential for practical applications.

## Conflicts of interest

The authors declare no conflict of interest.

## Supplementary Material

RA-009-C9RA02411E-s001
