# Supplementary material for: One-pot construction of Cu and O co-doped porous g-C3N4 with enhanced photocatalytic performance towards the degradation of levofloxacin
Source: RSC Adv. 2019 Jul 2;9(36):20633–42. doi: 10.1039/c9ra02411e (PMC9066014; doi:10.1039/c9ra02411e)
Supplement: RA-009-C9RA02411E-s001 [file RA-009-C9RA02411E-s001.pdf]

Electronic Supplementary Material (ESI) for RSC Advances.
This journal is © The Royal Society of Chemistry 2019

Supporting Information

One-pot construction of Cu and O co-doped porous g-C_3N_4 with enhanced photocatalytic performance towards the degradation of levofloxacin

Feng Li [a], Peng Zhu [b], Songmei Wang [c], Xiuquan Xu [b,*], Zijun Zhou [b], Chundu Wu [c,*]

[a] *Affiliated Hospital of Jiangsu University, Zhenjiang 212001, China*

[b] *School of Pharmacy, Jiangsu University, Zhenjiang 212013, China*

[c] *School of Environment and Safaty Engineering, Jiangsu University, Zhenjiang 212013, China.*

*Corresponding author.

E-mail address: xxq781026@ujs.edu.cn (X. X.); wucd@ujs.edu.cn (C. W.)

Experiment

The FT-IR spectrum of CN and Cu/O-PCN-3 were identified on a Fourier transform infrared spectrophotometer (Avater 370, Nicolet Co., USA). The sample about of 2.0 mg was evenly mixed with dried KBr powder and pressed into pellets for FT-IR determination over the wavelength range of 4000 - 400 cm^{-1}.

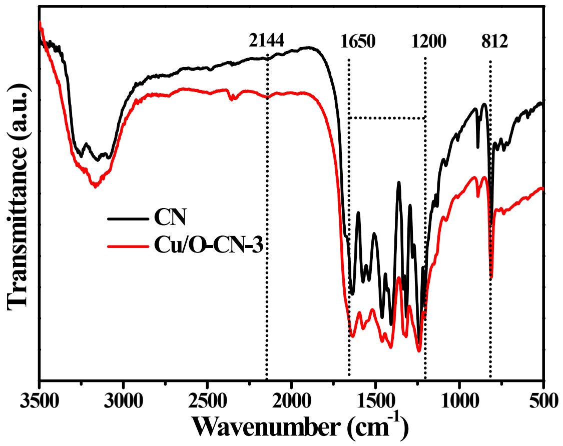

Fig. S1 FT-IR spectra of CN and Cu/O-PCN-3 nanomaterials.

The FT-IR spectra of CN and Cu/O-PCN-3 are given in Fig. S1. For CN and Cu/O-PCN-3 samples, a series of peaks ranging from 1200 to 1650 cm^{-1} are attributed to the typical stretching vibration of CN heterocycles, while the sharp peak located at 812 cm^{-1} is assigned to the vibrational mode of repeating triazine units, indicating that heptazine units are retained after Cu and O co-doping.[1] Compared with CN, an extra peak at 2144 cm^{-1} corresponds to the nitrile groups appeared in Cu/O-PCN-3,[2] which maybe result from the incomplete polymerization when adding $Cu(NO_3)_2·3H_2O$. Obviously, the peaks appears at about 576 and 630 cm^{-1} corresponds to the stretching vibration of Cu (Ⅱ)-O and Cu (Ⅰ)-O bonds are not detected in Cu/O-PCN-3. The result of FT-IR further confirms that Cu was ion-doped into the g-C_3N_4.[3]

Reference

1 H. L. Dou, L. Chen, S. H. Zheng, Y. P. Zhang and G. Q. Xu, *Mater. Chem. Phys.*, 2018, **214**, 482-488.

2 J. S. Hu, P. F. Zhang, W. J. An, L. Liu, Y. H. Liang, and W. Q. Cui, *Appl. Catal. B: Environ.*, 2019, 245, 130-142.

3 Y. L. Tian, B. B. Chang, J. Fu, B. C. Zhou, J. Y. Liu, F. N. Xi, and X. P. Dong, *J. Solid State Chem.*, 2014, **212**, 1-6.
